# Improving the Performance of Arsenene Nanoribbon Gate-All-Around Tunnel Field-Effect Transistors Using H Defects

**DOI:** 10.3390/nano14231960

**Published:** 2024-12-06

**Authors:** Shun Song, Lu Qin, Zhi Wang, Juan Lyu, Jian Gong, Shenyuan Yang

**Affiliations:** 1 State Key Laboratory of Superlattices and Microstructures, Institute of Semiconductors, Chinese Academy of Sciences, Beijing 100083, China; ssong@semi.ac.cn (S.S.); wangzhi@semi.ac.cn (Z.W.); 2 School of Physics and Technology, Inner Mongolia University, Hohhot 010021, China; qinlu@iet.cn (L.Q.); lvjuan@semi.ac.cn (J.L.); 3 College of Materials Science and Opto-Electronic Technology, University of Chinese Academy of Sciences, Beijing 101409, China

**Keywords:** arsenene nanoribbon, gate-all-around, field-effect transistors, defects

## Abstract

We systematically study the transport properties of arsenene nanoribbon tunneling field-effect transistors (TFETs) along the armchair directions using first-principles calculations based on density functional theory combined with the non-equilibrium Green’s function approach. The pristine nanoribbon TFET devices with and without underlap (UL) exhibit poor performance. Introducing a H defect in the left UL region between the source and channel can drastically enhance the ON-state currents and reduce the SS to below 60 mV/decade. When the H defect is positioned far from the gate and/or at the center sites, the ON-state currents are substantially enhanced, meeting the International Technology Roadmap for Semiconductors requirements for high-performance and low-power devices with 5 nm channel length. The gate-all-around (GAA) structure can further improve the performance of the devices with H defects. Particularly for the devices with H defects near the edge, the GAA structure significantly reduces the SS values as low as 35 mV/decade. Our study demonstrates that GAA structure can greatly enhance the performance of the arsenene nanoribbon TFET devices with H defects, providing theoretical guidance for improving TFET performance based on two-dimensional material nanoribbons through the combination of defect engineering and GAA gate structures.

## 1. Introduction

As Moore’s law approaches the end, it is impossible to continue the enhancement of the performance of traditional metal oxide semiconductor field-effect transistors (MOSFETs) by further decreasing the sizes. New materials, new mechanisms, and new structures have been extensively explored for new device technologies in the post-Moore era [1]. During the last decades, two-dimensional (2D) materials have been extensively investigated to design and fabricate electronic devices at the nanoscale [2,3,4,5] due to their ultra-thin atomic layer thickness and excellent physical properties [6,7,8]. Phosphorene features a suitable direct band gap, excellent carrier mobility, and remarkable anisotropy in its electronic and conductive characteristics [9,10]. The fabricated phosphorene devices exhibited excellent performance [10]. Moreover, the electronic and transport properties of phosphorene nanoribbons are nearly not influenced by edge defects [11]. However, the large-scale application to devices of phosphorene is hindered by its relatively low stability [12]. In contrast, layered arsenene, which shares similar electronic features with phosphorene, offers improved environmental stability, making it more suitable for practical applications [13,14]. Therefore, arsenene has attracted much interest as a promising channel material for nanoscale devices during the last few years. The arsenene field-effect transistor fabricated by Zhong et al. had an ON-state current of 1 × 10^−6^ A and a high $I_{\mathrm{ON}}/I_{\mathrm{OFF}}$ ratio of over 10^5^ [13]. Theoretical simulations by Qu et al. demonstrated that the arsenene MOSFETs possess a high $I_{\mathrm{ON}}/I_{\mathrm{OFF}}$ ratio and low power dissipation [15]. However, the subthreshold swing (SS) values of these devices were larger than the fundamental limit of 60 mV/decade for MOSFETs at room temperature [16]. With a band-to-band tunneling mechanism, the tunneling field-effect transistors (TFETs) have the potential to overcome the 60 mV/decade limit and achieve low OFF-state currents [17,18], but suffer from the small ON-state currents [19]. Theoretical researchers have predicted that the proper defects can improve the ON-state currents and decrease the SS of 2D material-based TFETs [20,21,22]. The fundamental reason is that the introduced mid-gap defect states can shorten the band-to-band tunneling path from the source to the channel at the ON-state [22]. Our previous studies have shown that the defects at the source–channel interface can greatly improve the performance of monolayer arsenene TFETs [23]. The SS can decrease to 50 mV/decade while the ON-state current is maintained at 3384 μA/μm with H defect.

With the decrease of device size, new structures, such as finned field-effect transistors, multi-gate transistors, and gate-all-around (GAA) transistors are promising strategies to reduce power consumption by increasing the gate control capability and decreasing the leakage current [24,25,26]. It is convenient to introduce GAA structure to nanoscale devices with finite width, such as nanowires and nanoplates [27,28]. Previous theoretical simulations on MOSFETs and TFETs based on 2D nanoribbons reported good performance of the devices [29,30,31]. However, there have been few theoretical reports on TFET devices based on 2D nanoribbons [32,33]. GAA TFETs with sub-5 nm channels using arsenene nanoribbons as channel material have not been explored in previous studies. Additionally, there is no research analyzing the arsenene nanoribbon GAA TFETs.

In this work, we comprehensively investigate the transport characteristics of arsenene nanoribbon TFETs along the armchair direction using first-principles calculations based on density functional theory (DFT) combined with the non-equilibrium Green’s function (NEGF) approach. The pristine nanoribbon TFETs exhibit insufficient ON-state currents and an excessively large SS value. Introducing an H defect in the left underlap (UL) region between the source and channel significantly enhances the ON-state currents and decreases SS to below 60 mV/decade. When the H defect is far away from the gate and/or at the center sites, the device ON-state currents are remarkably enhanced and meet the International Technology Roadmap for Semiconductors (ITRS) requirements for both high-performance (HP) and low-power (LP) devices. The performance of the devices with H defects can further be improved by GAA modulation. Particularly for those with H defects near the edge, the introduction of the GAA significantly decreases the SS values up to 35 mV/decade. Our findings demonstrate that GAA can significantly improve the performance of the arsenene nanoribbon TFETs with H defects, which can be used as a theoretical guide for optimizing the performance of TFETs based on 2D material nanoribbons through defects combined with GAA structure.

## 2. Computational Methods

The calculations of structural optimizations and transport characteristics in our study were performed using the Quantum ATK package based on DFT combined with the NEGF method [34]. We employed the generalized gradient approximation of Perdew−Burke−Ernzerof (PBE) for the exchange-correlation functionals [35]. We chose the double-zeta plus polarization orbitals as the basis set, and set the energy cutoff for real-space mesh as 45 Hartree. To study the properties of H defects on arsenene nanoribbons, we introduced one H atom to different adsorption sites on a 10-atom wide armchair arsenene nanoribbon. The armchair edge of the nanoribbon was passivated by hydrogen atoms and the supercell length along the periodic z direction was 17.84 Å. A vacuum thickness of 15 Å was introduced along the other two directions to reduce the interactions between adjacent nanoribbons under periodic boundary conditions. A 1 × 1 × 6 k-point grid was employed to sample the first Brillouin zone for structure relaxation and electronic calculation. The atomic coordinates were fully optimized until the force on each atom was less than 0.01 eV/Å. Due to the enhanced screening effects in FETs, GGA can accurately describe the exchange and correlation interactions in ultrathin devices [25,26,36]. Electron–electron interaction can be screened by the dielectric layer, especially for ultrathin 2D material-based devices. Furthermore, the carrier injection into the channel is increased when the device is working, significantly screening the electron–electron interaction. Therefore, GGA can give a reasonable bandgap for 2D materials in a device environment [25,26,36]. Additionally, we will use the GGA band gaps for later device simulations.

We constructed double gate or gate-all-around arsenene nanoribbon p-type TFET devices along the armchair transport direction, focusing on the influence of H defects and gate-all-around modulations on their transport properties. The H defect was introduced near the source/channel interface. A K-point grid of 1 × 1 × 106 was used for DFT self-consistent calculations of the devices. We adopted the ITRS 2013 standard to evaluate our devices. The ITRS version (with the shortest channel length of 5 nm) [1] is stricter than the latest International Roadmap for Devices and Systems (IRDS) 2022 version (with the shortest channel length of 12 nm) [37] and is more suitable for our sub-10-nm devices. In the following, “ITRS 2028” is referred to the ITRS 2013 edition for 2028 performance parameters. The equivalent oxide thickness (EOT) for each dielectric layer was 0.41 nm according to the ITRS 2028 standard, representing the thickness of silicon oxide required to produce the equivalent effect as the high-k dielectric material. Following the ITRS 2028 requirements, the source and drain bias voltage was fixed at $V_{\mathrm{ds}}$ = 0.64 V. The current $I\left(V_{\mathrm{ds}},V_{g}\right)$ was calculated using the Landauer–Büttiker formula [38]: $I\left(V_{\mathrm{ds}},V_{g}\right)=\frac{2e}{h}\int T\left(E,\left.V_{\mathrm{ds}},V_{g}\right)\right.\left[f_{L}\right.\left(E-\left.\mu_{L}\right)\right.-\left.f_{R}\left(E-\left.\mu_{R}\right)\right.\right]dE,$ where *V*_g_ is the gate voltage, $T\left(E,\left.V_{\mathrm{ds}},V_{g}\right)\right.$ is the transmission coefficient, and $f_{L}\left(E-\left.\mu_{L}\right)\right.$ and $f_{R}\left(E-\left.\mu_{R}\right)\right.$ are the Fermi–Dirac distribution functions of the left and right electrodes, respectively. For all the TFET devices, the n-type source and p-type drain electrodes were doped with a concentration of 2.07 × 10^11^ cm^−2^, while the channel remained intrinsic. Following the ITRS requirements, $V_{\mathrm{OFF}}$ is defined as the gate voltage with the OFF-state current $I_{\mathrm{OFF}}$ at 0.1 and 2 × 10^−5^ μA/μm for HP and LP devices, respectively. The ON-state current $I_{\mathrm{ON}}$ is defined as the current at the gate voltage of $V_{\mathrm{ON}}=V_{\mathrm{OFF}}+V_{\mathrm{ds}}$.

## 3. Results and Discussion

The 2D arsenene monolayer exhibits smaller carrier effective masses, higher carrier mobilities, and larger ON-state currents along the armchair direction compared to the zigzag direction [13,23]. Consequently, we focused on the arsenene nanoribbon TFET devices along the armchair direction. The edge of the nanoribbon is passivated by H atoms, as shown in Figure S1a in the Supplementary Information (SI). As seen from Figure S1b, the bandgap of the nanoribbon is highly dependent on its width. The 10-atom width nanoribbon has a width of 18.73 Å and a moderate indirect bandgap of 1.33 eV, which are suitable for nanoscale TFET devices. We, thus, choose this nanoribbon to construct the TFET devices in the following study.

**Figure 1:**
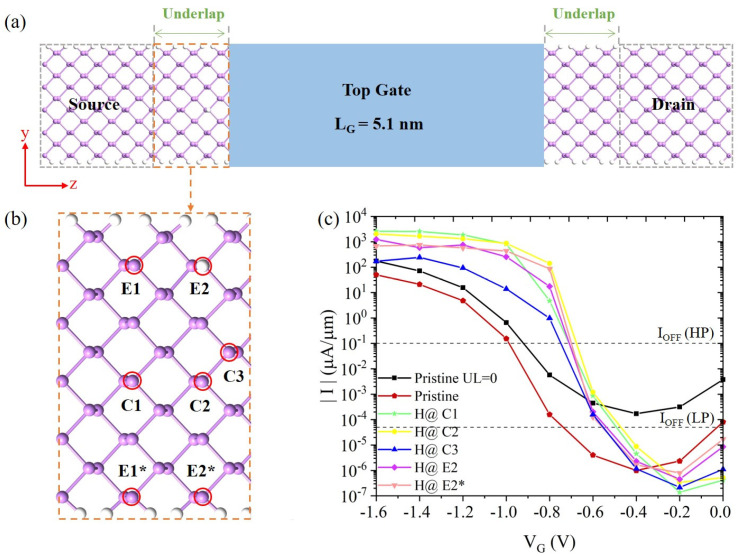
(**a**) Schematics of the arsenene nanoribbon double gate TFET device along the armchair direction with UL = 1 nm. (**b**) One H defect is introduced in the left underlap at the source–channel interface denoted by an orange rectangle at different adsorption sites. The H defect is adsorbed above the As atoms marked in the red circles. (**c**) Current-voltage characteristics of the arsenene nanoribbon TFET devices with and without defect and UL.

Figure 1a shows the schematic of the 5.1 nm arsenene nanoribbon double-gate TFET device along the armchair transport direction. To enhance the device performance, a UL structure is introduced on both ends of the channel, which decreases the impacts of the source and drain on the channel and reduces leakage current [26]. However, an excessively long UL would deteriorate the gate controllability [26,39]. We choose a moderate UL length of 1 nm. As shown in Figure 1c and Table S1, the device without an UL exhibits a minimal OFF-state current of 1.71 × 10^−4^ μA/μm and a low ON-state current of less than 200 μA/μm, failing to satisfy the ITRS requirements for HP (900 μA/μm) and LP (295 μA/μm) devices. However, the SS value of 95 mV/decade is relatively large. By introducing a 1nm UL, the device has a longer tunneling path (see Figure S2a,b). As a result, the OFF-state current is reduced below 2 × 10^−5^ μA/μm and the SS is improved to 67 mV/decade. However, the ON-state current is further reduced to below 100 μA/μm. Therefore, the pristine arsenene nanoribbon TFET devices with and without UL do not satisfy the ITRS requirements for both HP and LP devices.

Our previous study demonstrated that introducing an H defect near the source–channel interface can greatly enhance the ON-state currents in monolayer arsenene TFET devices [23]. Inspired by these findings, we introduced H defects in the left UL region between the source and channel, aiming to increase the ON-state currents and improve the SS of the nanoribbon devices. We find that the adsorption sites of the H atom significantly influence the performance of the devices. As shown in Figure 1b, the center site, the next to edge site, and the edge site were named as the C, E, and E* sites, respectively. We considered three distances of the H atoms with respect to the left side of the gate along the z direction (7.82, 3.38, and 1.39 Å), denoted as 1, 2, and 3 in Figure 1b, respectively. The devices with H far away from the gate (C1, E1 and E1* sites) exhibited the best performance, meeting the ITRS requirements for both HP and LP devices with a 5 nm channel length (Table S1). For instance, the ON-state currents with H at C1 site were 2538 and 1860 μA/μm for HP and LP devices, respectively. As the H defect moves closer to the gate, the ON-state current gradually decreases. When the H atom is adsorbed at C3 site, the ON-state current is significantly reduced to 243 and 94 μA/μm for HP and LP devices, respectively, failing to meet the ITRS requirements. We also found that the devices with H at the center performed better than those with H near the edge. With an H defect at the C2 site, the ON-state currents were 1655 and 859 μA/μm for HP and LP devices, respectively, meeting the ITRS requirements. However, when the H atom is adsorbed at the E2 and E2* sites, the TFETs have lower ON-state currents and can only be used as LP devices (Table S1).

**Figure 2:**
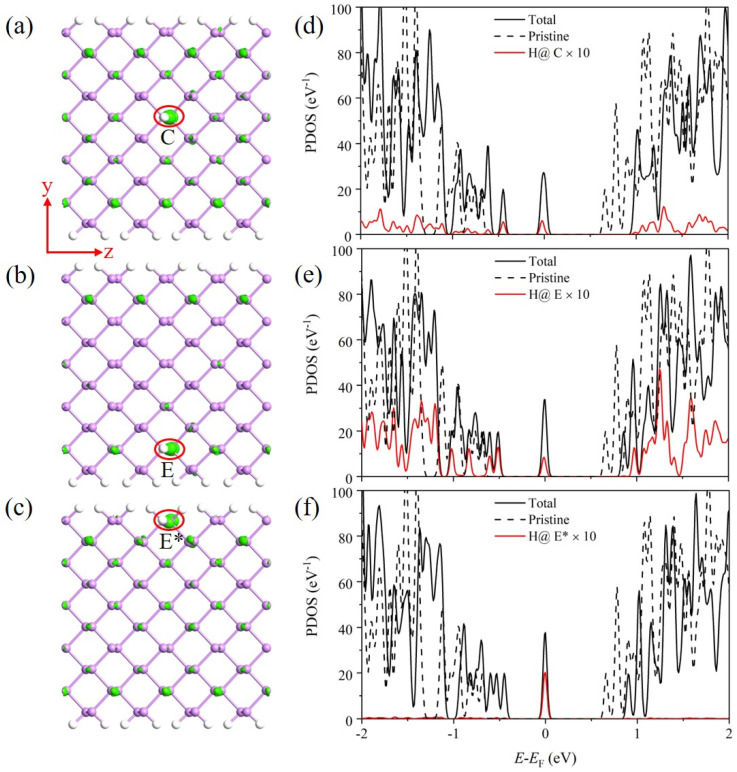
Partial charge densities and PDOSs of arsenene nanoribbons with an H defect adsorbed at (**a**,**d**) C, (**b**,**e**) E, and (**c**,**f**) E* sites. The isosurface value of the partial charge density is 0.25 Å^−3^. The Fermi level *E*_F_ is set as energy zero. The density of states of the pristine arsenene nanoribbon is presented using dashed lines for comparison. The PDOSs of the H defects are magnified by 10 times for clarity.

The influence of an H defect on the devices is closely related to the properties of the defect state. A larger broadening means more tunnelling channels, and a wider distribution means the defect state can better assist the carrier tunnelling from the source to the channel. Figure 2 shows the partial charge densities and the projected density of states (PDOSs) of the arsenene nanoribbon with H defects at different sites. The adsorption of an H atom introduces a defect state in the band gap. The defect state has maximum broadening when H is adsorbed at the C site, as shown in Figure 2d. As the H atom moves to the edge sites (E and E*), the broadening of the defect state gradually decreases, as shown in Figure 2e,f. Figure 2a–c show that the defect state at the C and E* sites is more delocalized compared to the E site, which could be more favorable for the carrier tunnelling between the source and the channel. A previous study on graphene nanoribbon-based FETs also reported that strongly localized bandgap states make negligible contributions to transport [40].

**Figure 3:**
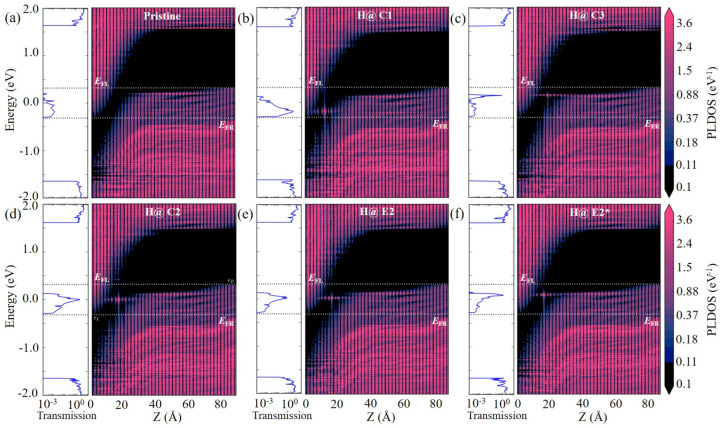
PLDOSs of the arsenene nanoribbon TFET devices at HP ON-states with and without H defects. (**a**) Pristine device without H defect. (**b**) H defect at C1 site. (**c**) H defect at C3 site. (**d**) H defect at C2 site. (**e**) H defect at E2 site. (**f**) H defect at E2* site. The UL is chosen to be 1 nm.

The better performance of the arsenene nanoribbon TFET devices with H at the center can be understood by the broadening of the H defect states. Figure 3 shows the projected local density of states (PLDOSs) of the nanoribbon devices at the ON-state. Compared to the pristine device, the H defect states significantly reduce the tunneling path from the source conduction band minimum (CBM) to the channel valence band maximum (VBM), leading to an increase in ON-state currents. As seen from Figure 3d–f, the H defect at the C2 site has a larger broadening than at E2 and E2* sites. This provides more tunnelling channels for carriers and results in a larger ON-state current at the C2 site. Similarly, the device with H at the C1 site has a larger ON-state current than those with H at the E1 and E1* sites. The influence of distance between the H defect and the gate is also found to be mainly related to the broadening of the defect state. Taking C1, C2, and C3 sites as examples, as the H defect moves from the C1 to C3 sites, the energy of the defect state shifts up towards the VBM of the channel, as shown in Figure 3b–d. Since there is no channel state above VBM that can couple with the H defect states, the broadening of the defect state becomes smaller as H moves close to the gate, resulting in the decreasing ON-state currents from the C1 to C3 sites.

The introduction of an H defect also improves the switching speed from the OFF- to ON-states. As seen in Figure S3, the gate voltage *V*_g_ greatly enhances the spatial distribution of the defect state at −0.8 V, and the rapid increase of the current from −0.6 to −0.8 V gives the steepest SS. As discussed earlier, the defect state with H at the E2 site is more localized compared to the C2 and E2* sites. This is also the case at *V*_g_ = −0.8 V (Figure S3) and, thus, the device has a larger SS than the devices with H at the C2 and E2* sites. The SS value also depends on the distance between the H defect and the gate. As shown in Figure S3, the defect state at the C2 site is within the tunneling window at *V*_g_ = −0.8 V, while the defect state at the C1 site is just near the tunneling window. Therefore, the device with H at the C1 site has a smaller current and a larger SS. This indicates that the control of the gate on the defect state would decrease if the defect was too far from the gate, which is not favorable for a steep SS.

From the current-voltage characteristics in Figure 1c, the introduction of the H defect could improve the switching speed from the OFF- to ON-states. The calculated SS values of all the devices with H defects are below the thermal limit of 60 mV/decade (Table S1). The gate voltage not only shifts the energy levels of the channel, but also enhances the spatial distribution of the defect states. As shown in Figure S3d–f, the H defect states are quite localized along the z direction (the transport direction) at *V*_g_ = −0.6 V, regardless of the adsorption sites. Therefore, all the devices have low currents at *V*_g_ = −0.6 V. As the negative gate voltage increases to *V*_g_ = −0.8 V (Figure S3a–c), the defect state becomes quite delocalized along the z direction and, thus, can effectively assist the tunneling from the source to the channel. The rapid increase of the current from −0.6 to −0.8 V gate voltage gives the steepest SS. As discussed earlier, the defect state with H at the E2 site is more localized than at the C2 and E2* sites. This is also the case at *V*_g_ = −0.8 V, as seen in Figure S4a. Therefore, the device with H at the E2 site has a smaller current at *V*_g_ = −0.8 V. Its SS value (41 mV/decade) is also larger than the devices with H at the C2 and E2* sites (40 and 35 mV/decade). Although the device with an H atom at the C1 site has a higher ON-state current than that at the C2 site, it has a worse SS value of 56 mV/decade. As shown in Figure S3a, when H is at the C1 site, the defect state is just near the tunneling window at *V*_g_ = −0.8 V. On the other hand, the defect state at the C2 site enters the tunneling window, as shown in Figure S3b. Thus, the device with H at the C2 site has a larger current at *V*_g_ = −0.8 V and a better SS.

**Figure 4:**
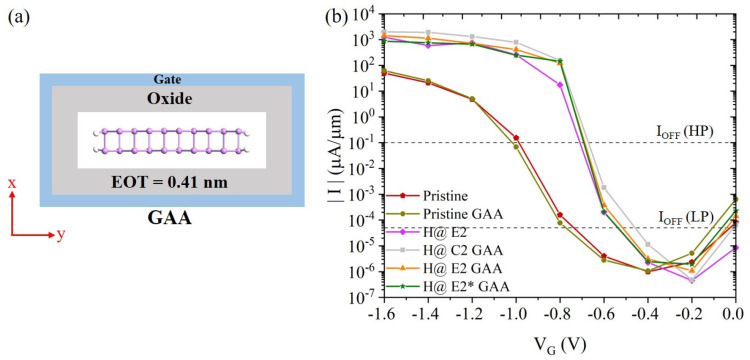
(**a**) Schematics of the cross-section of the arsenene nanoribbon GAA TFET device along the armchair direction. (**b**) Current-voltage characteristics of the arsenene nanoribbon TFET devices with and without H defects and GAA. The UL is chosen to be 1 nm.

The GAA gate structure provides better electrostatic control of the channel that can effectively suppress the short-channel effects [41,42]. Here we introduce GAA to improve the performance of arsenene nanoribbon devices. Figure 4a shows the schematic of the cross-section of the GAA devices. As seen in Figure 4b and Table S1, the introduction of GAA just slightly enhances the ON-state current of the pristine TFET device. On the other hand, GAA can significantly improve the performance of devices with H defects. Particularly for the device with an H defect at the E2 site, the GAA significantly increases the ON-state current from 585 to 1126 μA/μm, and reduces SS from 41 to 35 mV/decade, causing the GAA device to be used as an HP device. In other words, the GAA devices with H defects at the center and near the edge can satisfy the ITRS requirements for HP and LP devices, loosening the restrictions on the H defect sites. However, if the H atom is adsorbed at the edge E2* site, the ON-current of the GAA device is still not high enough for the HP device. Our calculations show that with GAA modulation, H defects at several positions can improve the device performance to meet the ITRS standards, greatly releasing the strict requirement for H positions. This would help to fabricate the devices in experiments or even in industry.

**Figure 5:**
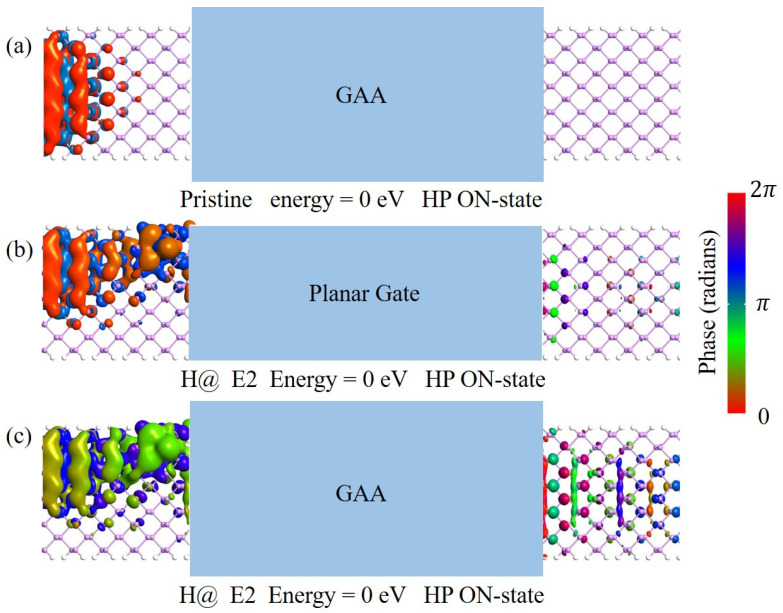
Transmission eigenstates of the arsenene nanoribbon TFET HP devices at the ON-state and at an energy of 0 eV. (**a**) GAA device without H defect. (**b**) Planar gate device with H defect at the E2 site. (**c**) GAA device with H defect at the E2 site.

The significant improvement of the device performance with H at the E2 site through GAA modulation can be understood by the transmittance eigenstates of the devices at the ON-state shown in Figure 5. A similar approach was used to analyze the transport properties of graphene nanoribbon-based field effect transistors in previous studies [43]. For the GAA device without an H defect, the eigenstates injected from the source at 0 eV are mainly localized at the left electrode, with negligible distribution at the right electrode, as shown in Figure 5a. This indicates that the eigenstates can hardly reach the right electrode through the channel. For the planar gate device with an H defect, some eigenstates can reach the right electrode through the channel with the help of the defect state, as shown in Figure 5b. GAA could enhance the defect-assisted tunnelling from the source to the channel. For the GAA device with an H defect, as shown in Figure 5c, the injected eigenstate wavefunction at the ON-state is distributed over a large region at both the left and the right electrodes.

The PLDOSs shown in Figure S4 further confirm the improvement with the H defect at the E2 site. Compared to the planar device, the defect state of the GAA device becomes more delocalized at *V*_g_ = −0.8 V. Therefore, the current of the GAA device increases more rapidly than in the planar device, and SS is improved from 41 to 35 mV/decade. In contrast, the defect states of the planar devices with H at the C2 and E2* sites are already quite delocalized, and the GAA structure just slightly improves the currents and SS values. We found a similar improvement of the SS value from 56 to 42 mV/decade for the device with an H defect at the E1 site. In other words, when the H defect is near the edge (E1 and E2 sites), GAA modulation can improve the control over the defect state by increasing its spatial delocalization, leading to a more rapid increase in current and a better SS value.

## 4. Conclusions

In summary, we have systematically investigated the transport properties of TFETs based on armchair arsenene nanoribbons using first-principles calculations and quantum transport simulations. The pristine nanoribbon TFET exhibits poor performance, with ON-state currents below 200 μA/μm and a large SS value of 95 mV/decade. By introducing a 1nm UL, the device SS is reduced to 67 mV/decade, but the ON-state current is further reduced to below 100 μA/μm. Introducing an H defect in the left UL region between the source and channel can greatly improve the ON-state currents and reduce the SS below 60 mV/decade. Desirable performance can be achieved when the H defect is positioned away from the gate and/or at central C sites, meeting ITRS requirements for HP and LP devices. Further improvement is realized with a GAA structure, particularly for a near-the-edge H defect, where SS values are reduced to as low as 35 mV/decade and ON-state currents can reach 1126 μA/μm. These findings provide theoretical insights into optimizing 2D material-based TFETs through the combination of defect engineering and GAA gate structures in the post-Moore era.

## Data Availability

The data that support the findings of this study are available from the corresponding author upon reasonable request.

## Supplementary Materials

The following supporting information can be downloaded at: https://www.mdpi.com/article/10.3390/nano14231960/s1, Figure S1: 10-atom width H-passivated armchair arsenene nanoribbons and arsenene nanoribbon band gap as a function of nanoribbon width; Figure S2: PLDOSs of arsenene nanoribbon TFET devices at the OFF-state; Figure S3: PLDOSs and transmission at the gate voltage of −0.6 and −0.8 V for arsenene nanoribbon TFET devices with H defects; Figure S4: PLDOSs of arsenene nanoribbon TFET devices with different gate structures at gate voltage of −0.8 V; Table S1: summary of the performance of arsenene nanoribbon TFET devices with and without defects and GAA.
