# Supplementary material for: Improving the Performance of Arsenene Nanoribbon Gate-All-Around Tunnel Field-Effect Transistors Using H Defects
Source: Nanomaterials (Basel). 2024 Dec 6;14(23):1960. doi: 10.3390/nano14231960 (PMC11643083; doi:10.3390/nano14231960)
Supplement: Supplementary file 1 [file nanomaterials-14-01960-s001.zip › nanomaterials-3317203-supplementary.pdf]

Supplementary Materials for

Improving the performance of arsenene nanoribbon gate-all-around tunnel field-effect transistors by H defects

Shun Song,[1] Lu Qin,[2] Zhi Wang,[1] Juan Lyu,[2] Jian Gong,[2,*] and Shenyuan Yang[1,3,*]

[1]State Key Laboratory of Superlattices and Microstructures, Institute of Semiconductors, Chinese Academy of Sciences, Beijing 100083, P. R. China

[2]School of Physics and Technology, Inner Mongolia University, Hohhot 010021, P. R. China

[3]College of Materials Science and Opto-Electronic Technology, University of Chinese Academy of Sciences, Beijing 101409, P. R. China

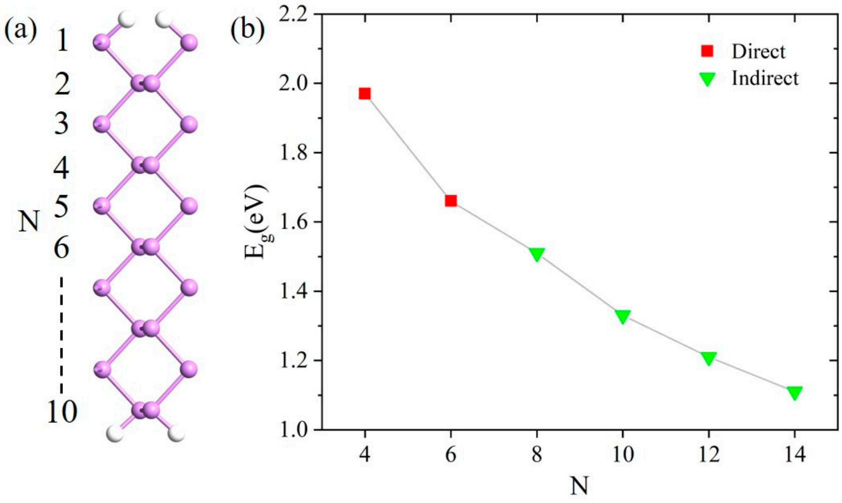

Figure S1. (a) 10-atom width H-passivated armchair arsenene nanoribbons. N denotes the number of atomic lines in the nanoribbon width. (b) Arsenene nanoribbon band gap as a function of the width.

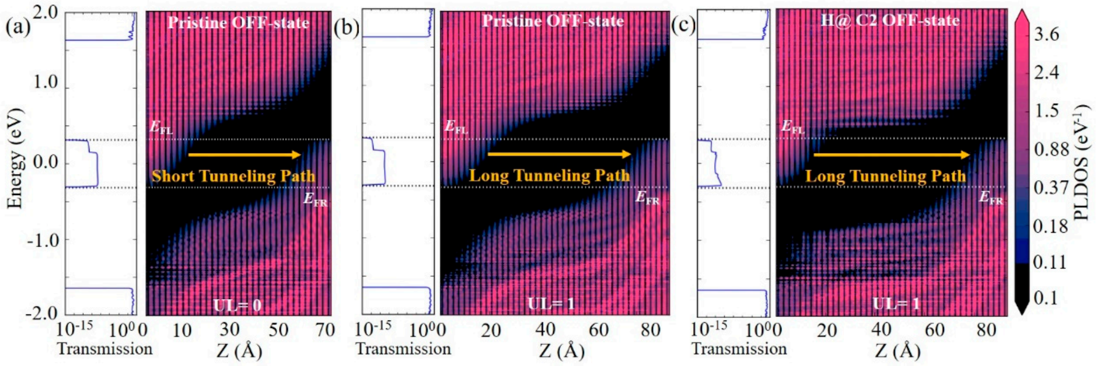

Figure S2. PLDOSs and transmission for arsenene nanoribbon TFET devices at the OFF-state. (a) Pristine device without defect and UL. (b) Pristine device without defect and the UL is chosen to be 1 nm. (c) Device with H defect at the C2 site and the UL is chosen to be 1 nm.

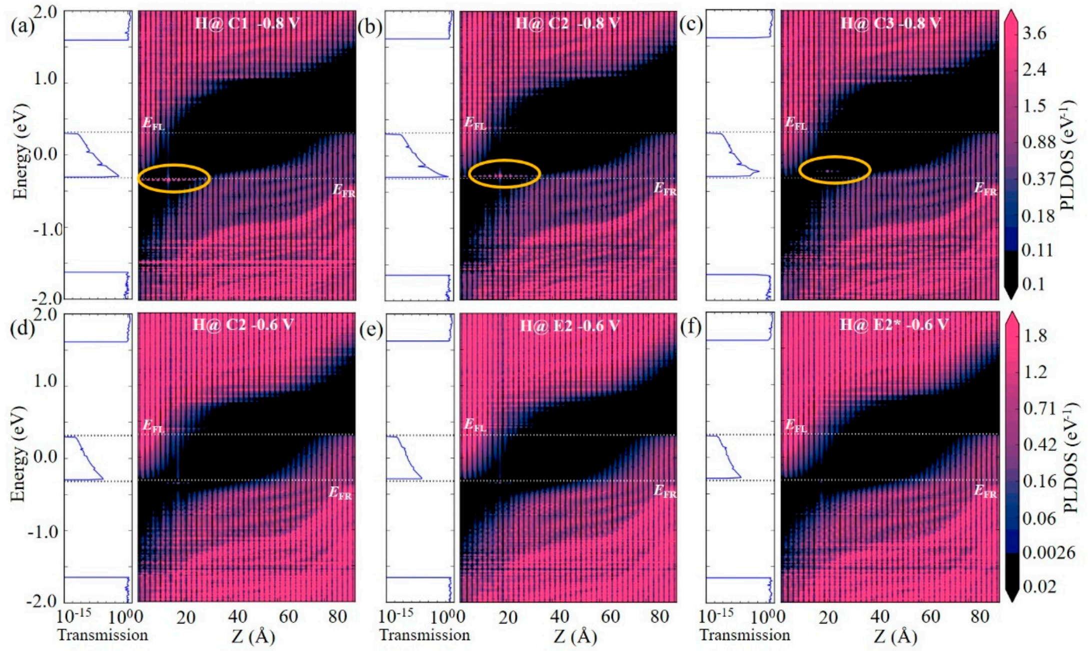

Figure S3. PLDOSs and transmission for arsenene nanoribbon TFET devices with H defects at V_g =-0.6 and -0.8 V. (a) H defect at the C1 site at V_g=-0.8V. (b) H defect at the C2 site at V_g=-0.8V. (c) H defect at the C3 site at V_g=-0.8V. (d) H defect at the C2 site at V_g=-0.6V. (e) H defect at the E2 site at V_g=-0.6V. (f) H defect at the E2* site at V_g=-0.6V. The UL is chosen to be 1 nm.

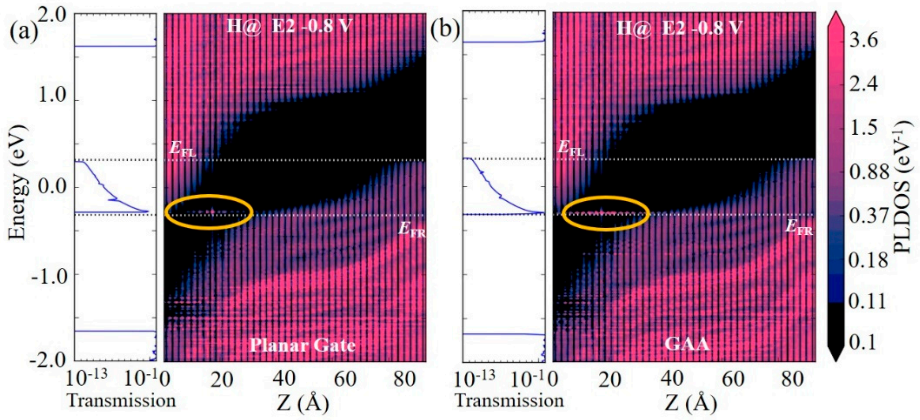

Figure S4. PLDOSs and transmission for arsenene nanoribbon TFET devices with different gate structures at V_g =-0.8V. (a) Planar gate device with H defect at E2 site. (b) GAA device with H defect at E2 site.

Table S1 Summary of the performance of arsenene nanoribbon TFET devices with and without defects and GAA. The ITRS 2028 targets for HP and LP devices at the 5 nm technology node are presented for comparison. The units of SS and current are mV/decade and $\mu A/\mu m$, respectively.

		planar		GAA	
devices	type	SS	I_{ON}	SS	I_{ON}
pristine without UL	HP	95	174	-	-
	LP			-	-
pristine	HP	67	50	67	62
	LP		21		25
C1	HP	56	2538	-	-
	LP		1860	-	-
C2	HP	40	1655	40	1897
	LP		859		783
C3	HP	52	243	-	-
	LP		94	-	-
E1	HP	56	1532	46	1718
	LP		1237		1305
E1*	HP	42	1314	45	1303
	LP		682		1028
E2	HP	41	585	35	1126
	LP		746		712
E2*	HP	35	745	34	751
	LP		574		662
ITRS 2028	HP	-	900	-	900
	LP	-	295	-	295
